# Trehalose Augments Neuron Survival and Improves Recovery from Spinal Cord Injury via mTOR-Independent Activation of Autophagy

**DOI:** 10.1155/2021/8898996

**Published:** 2021-07-10

**Authors:** Kailiang Zhou, Huanwen Chen, Huazi Xu, Xiaofeng Jia

**Affiliations:** ^1^Department of Orthopaedics, The Second Affiliated Hospital and Yuying Children's Hospital of Wenzhou Medical University, Zhejiang 325027, China; ^2^Department of Neurosurgery, University of Maryland School of Medicine, Baltimore, MD 21201, USA; ^3^Department of Orthopaedics, University of Maryland School of Medicine, Baltimore, MD 21201, USA; ^4^Department of Anatomy and Neurobiology, University of Maryland School of Medicine, Baltimore, MD 21201, USA; ^5^Department of Biomedical Engineering, The Johns Hopkins University School of Medicine, Baltimore, MD 21205, USA; ^6^Department of Anesthesiology and Critical Care Medicine, The Johns Hopkins University School of Medicine, Baltimore, MD 21205, USA

## Abstract

Spinal cord injury (SCI) is a major cause of irreversible nerve injury and leads to serious tissue loss and neurological dysfunction. Thorough investigation of cellular mechanisms, such as autophagy, is crucial for developing novel and effective therapeutics. We administered trehalose, an mTOR-independent autophagy agonist, in SCI rats suffering from moderate compression injury to elucidate the relationship between autophagy and SCI and evaluate trehalose's therapeutic potential. 60 rats were divided into 4 groups and were treated with either control vehicle, trehalose, chloroquine, or trehalose + chloroquine 2 weeks prior to administration of moderate spinal cord crush injury. 20 additional sham rats were treated with control vehicle. H&E staining, Nissl staining, western blot, and immunofluorescence studies were conducted to examine nerve morphology and quantify autophagy and mitochondrial-dependent apoptosis at various time points after surgery. Functional recovery was assessed over a period of 4 weeks after surgery. Trehalose promotes autophagosome recruitment via an mTOR-independent pathway, enhances autophagy flux in neurons, inhibits apoptosis via the intrinsic mitochondria-dependent pathway, reduces lesion cavity expansion, decreases neuron loss, and ultimately improves functional recovery following SCI (all *p* < 0.05). Furthermore, these effects were diminished upon administration of chloroquine, an autophagy flux inhibitor, indicating that trehalose's beneficial effects were due largely to activation of autophagy. This study presents new evidence that autophagy plays a critical neuroprotective and neuroregenerative role in SCI, and that mTOR-independent activation of autophagy with trehalose leads to improved outcomes. Thus, trehalose has great translational potential as a novel therapeutic agent after SCI.

## 1. Introduction

Spinal cord injury (SCI) is a major cause of significant central nerve system (CNS) injury and leads to irreversible tissue loss and neurological dysfunction [1, 2]. In the United States alone, approximately 273,000 individuals suffer from SCI, with almost 12,000 new incidents occurring each year [3]. Despite obvious clinical need, current treatments and management strategies are far from satisfactory. To develop effective therapeutic interventions, understanding the detailed biological mechanisms of SCI is critical. The pathological process of SCI is initiated by primary injury, which often involves direct mechanical tissue damage. Then, a cascade of detrimental events collectively termed secondary injury follows [4], including local ischemia, excitotoxic chemical release, calcium overload, free radical species generation, inflammatory reaction, and neural cell death [5, 6]. Given the complex pathophysiology of acute CNS injuries such as SCI, thorough investigation of regulatory mechanisms involving neuron death is crucial for the development of novel and effective therapeutics [7, 8].

Autophagy, a major pathway for bulk cytosolic degradation, efficient turnover under stress, and cellular homeostasis [9, 10], is widely hypothesized to be an important player in a variety of diseases. In terminally differentiated cells such as neurons, autophagy plays a particularly critical role, as pathological organelles and proteins cannot be redistributed through cellular mitosis and proliferation [11, 12]. Studies have shown that augmenting autophagy is beneficial for chronic CNS diseases that involve chronic intracellular accumulation of toxic materials such as Alzheimer's disease, Huntington's disease, and amyotrophic lateral sclerosis [13–15]. Damaged proteins and organelles can also accumulate in neurons following acute CNS injuries such as SCI; thus, we hypothesize that stimulating autophagy may also be beneficial in acute injuries such as traumatic SCI (TSCI). While various studies have attempted to elucidate the role of autophagy in TSCI [9, 16–18], results remain controversial. Rapamycin, a classic and commonly used autophagy activator that inhibits the mechanistic target of rapamycin (mTOR) pathway, has largely been the only agent used in TSCI research to augment autophagy activation and has produced contradictory results [16, 18]. This is in part due to unfavorable systemic side-effects of mTOR-dependent autophagy activators in these TSCI studies [19, 20]. These agents, such as rapamycin (a stereotypical autophagy agonist), are known to regulate a wide range of cellular processes beyond autophagy including growth, metabolism, and inflammation [21, 22]. In acute CNS injuries, mTOR inhibitors are particularly problematic since they have known antiangiogenic effects [23] that can restrain neuroregeneration and impede functional recovery [24, 25]. Thus, in order to fully elucidate the role of autophagy in SCI, agents that act via an mTOR-independent pathway more specific to autophagy must be explored.

Trehalose (TRE), a nonreducing natural disaccharide (a,a-1,1-glucoside) that exerts important functions that protect proteins and stabilize membranes under stress conditions in plants, insects, microorganisms, and invertebrates [26], has emerged as an exciting new agent capable of activating autophagy via an mTOR-independent mechanism [27]. Recent literature suggests that trehalose provides potent neuroprotection in animal models of chronic CNS diseases such as Huntington's disease [28], Alzheimer's disease [29], and amyotrophic lateral sclerosis [30], suggesting that trehalose may not only be an effective tool to study how autophagy impacts SCI injury and recovery, it may also be an effective therapeutic agent for SCI. Recently, some reports have suggested that trehalose may improve outcomes following SCI in animal models; however, whether these benefits are related to autophagy is unknown [31–34]. Thus, for the first time, we utilized the novel autophagy activator trehalose via oral administration to investigate the role of mTOR-independent activation of autophagy in a translational animal model for compression SCI. Furthermore, we assessed trehalose's therapeutic potential in this common and devastating disease.

## 2. Materials and Methods

### 2.1. Reagents and Antibodies

Trehalose (C_12_H_22_O_11_·2H_2_O, HPLC > 99%, Cat. No. T9531) and chloroquine (C_18_H_26_C_l_N_3_·2H_3_PO_4_, HPLC > 98%, Cat. No. C6628) were acquired from Sigma-Aldrich (St. Louis, MO, USA). The H&E staining kit (Cat. No. 7111 and 7211) and the Pierce bicinchoninic acid (BCA) kit (Cat. No. 23225) were purchased from Thermo Fisher Scientific (San Jose, CA, USA). Primary antibodies against mTOR (Cat. No. 2983), phospho-mTOR (Cat. No. 2971), P70S6K (Cat. No. 2708), phospho-P70S6K (Cat. No. 9208), Beclin1 (Cat. No. 3738), ubiquitin (Cat. No. 3936), cytochrome c (Cat. No. 4272), Bax (Cat. No. 2772), PARP (Cat. No. 9542), and cleaved CASP3 (Cat. No. 9664) were acquired from Cell Signaling Technology (Beverly, MA, USA). Primary antibodies against cathepsin D (CTSD, Cat. No. sc-6486) and horseradish peroxidase- (HRP-) conjugated donkey-anti-goat IgG antibody (Cat. No. sc-2020) were obtained from Santa Cruz Biotechnology (Santa Cruz, CA, USA). Primary antibodies against LC3B (Cat. No. L7543), p62/SQSTM1 (Cat. No. P0076), *β*-actin (Cat. No. A5441), and DAPI staining solution (Cat. No. D9542) were obtained from Sigma-Aldrich (St. Louis, MO, USA). HRP-conjugated goat-anti-rabbit IgG antibodies (Cat. No. C50617-01) and goat-anti-mouse IgG antibodies (Cat. No. C50814-01) were obtained from LI-COR Biotechnology (Bad Homburg, Germany). Primary antibodies against NeuN (Cat. No. MAB377), fluorescein isothiocyanate- (FITC-) conjugated goat-anti-mouse IgG antibodies (Cat. No. AP181F), and the enhanced chemiluminescence (ECL) reagent (Cat. No. WBKLS0100) were purchased from Millipore Corporation (Bedford, MA, USA). FITC-conjugated donkey-anti-goat IgG antibodies (Cat. No. A16006) and tetramethylrhodamine isothiocyanate- (TRITC-) conjugated goat-anti-rabbit IgG antibodies (Cat. No. A24542) were acquired from Thermo Fisher Scientific (San Jose, CA, USA).

### 2.2. Animals

Adult Wistar rats, male, weighing 275-300g, were purchased from Charles River (Wilmington, MA). All animals were maintained according to National Institutes of Health (NIH) guidelines, and all experimental protocols were approved by the Institutional Animal Care and Use Committee (IACUC) of the University of Maryland School of Medicine. Every effort was made to reduce the total number of rats and their discomfort and pain. 80 rats were randomly divided into five groups: 20 rats in the control group, 20 rats in the SCI group, 5 rats in the SCI+CQ (chloroquine) group, 20 rats in the SCI+TRE (trehalose) group, and 15 rats in the SCI+TRE+CQ group. For two weeks before surgical procedures, all rats were housed in a room with a 12 : 12 h light/dark cycle and free access to regular food and water.

### 2.3. SCI Model

The surgical procedure of SCI has been described as we published [5, 35]. In brief, rats were anesthetized with 1.5% isoflurane delivered by a vaporizer before the operation. During the procedure, the temperature of the rats was monitored and maintained at 37°C with a constant heating pad. Laminectomy was performed at the level of T9-T10 to expose the dorsal cord surface with the dura intact. The intact spinal cord was subjected to the compression induced at the T9-T10 level by a vascular clip (15g force; Oscar, China) for 1 min. The rats in the control group received the same procedures of laminectomy, but without compression. Then, muscle and skin were sutured in layers with 4-0 silk and needle. After the operation, rats received rehabilitation daily: each SCI rat underwent manual urinary bladder emptying three times daily until bladder function returned and received rehabilitation massages (passive mobilization of the paralyzed legs) five times every day. All rats were euthanized with an overdose of isoflurane. On day 3, 5 rats in each group were sacrificed for Western blot analysis, and 5 rats in the control, SCI, SCI+TRE, and SCI+TRE+CQ groups were sacrificed for immunofluorescence. On day 7, 5 rats each from the control, SCI, SCI+TRE, and SCI+TRE+CQ groups were sacrificed for HE and Nissl staining. On days 1, 3, 7, 14, and 28, 5 rats in the control, SCI, and SCI+TRE groups, respectively, were tested using the Basso, Beattie, and Bresnahan (BBB) rating scale.

### 2.4. Drug Administration

Providing trehalose via drinking water is a common practice in rat models studying trehalose's effects [30, 36, 37]. Before the SCI surgical operation, rats in the SCI+TRE group were treated with regular drinking water containing trehalose, which was diluted into a final concentration of 2% (*w*/*v*) [36], for 7 days successively. To standardize normal daily water intake in rats, each rat was raised in a single cage and given a limited daily ration of drinking water (35 ml). Rats in the SCI+CQ group received a dose of 25 mg/ml CQ-saline solution (50 mg/kg, i.p.) for 7 consecutive days. Rats in the SCI+TRE+CQ group received 25 mg/ml CQ-saline solution (50 mg/kg, i.p.) in addition to the same trehalose administration with the SCI+TRE group for 7 consecutive days. The dose and time of CQ administration were decided according to previous studies in SCI [38]. In the control group and the SCI group, rats were provided regular drinking water and an equal dose of saline solution (vehicle control). Following the surgical operation, rats in each group were housed individually in standard experimental cages and were, respectively, given the same agent and control treatment as the preoperative treatments for 3 days successively.

### 2.5. Behavioral Study

The open field testing of the Basso, Beattie, and Bresnahan (BBB) rating scale for rats was performed to assess the severity of paralysis and motor dysfunction due to spinal cord injury. As described previously [39], rats (*n* = 5 for each group) in the control, SCI, and SCI+TRE groups were trained daily to adapt to the open field, a plastic wading pool with a smooth floor (900 mm in diameter) and 70 mm high walls, two weeks before the surgery. On day 1, day 3, day 7, day 14, day 21, and day 28 after SCI, rats in each group were laid in the center of the field and their crawling capability was observed for 5 min. During the evaluation, obtaining the BBB score of each rat was performed according to the BBB score protocol [39]. In brief, the BBB score ranges from 0 point, which is indicative of complete paralysis, to 21 points, which indicates normal locomotion. BBB scores were obtained by two independent examiners who were blind to each group.

### 2.6. Hematoxylin and Eosin (H&E) Staining

On day 7 after SCI, rats in each group (*n* = 5) were deeply anesthetized with isoflurane and euthanized by transcardiac perfusion with ice-cold 100 mM phosphate-buffered saline (PBS, PH 7.4) followed by addition of 4% (*w*/*v*) paraformaldehyde (PFA) in PBS. The rostral and caudal spinal cord segments (1 mm in length, 4 mm far from the epicenter, respectively) and epicenter segment (1 mm in length) were separated and postfixed in PFA overnight. Next, all samples were washed and embedded in paraffin wax. The paraffin sections (5*μ*m) were cut and mounted on gelatin-coated slides for H&E staining. H&E staining protocol was performed as previously described [40, 41]. The H&E staining slides were observed by a light microscope (Nikon, Japan). To quantify damage severity to the spinal cord, the percent of cavity area in the total area of 10 slices per spinal cord segment were autocalculated and averaged in each group with ImageJ software (version 1.41.0, National Institutes of Health, USA).

### 2.7. Nissl Staining for Neuron Counting

Slides from rostral, caudal, and epicenter spinal cord segments were deparaffinized in xylene and rehydrated through a graded set of ethanol. Next, the slides were incubated in 1% cresyl violet acetate at room temperature for 30 min and washed twice with PBS. Then, the slides were visualized at 400x magnifications under a light microscope (Nikon, Japan). Nissl-positive cells were visualized to define the neurons in the anterior horns. The anterior horns at both sides of 10 slices per spinal cord segment were examined, and the number of neurons was manually enumerated and averaged in a strictly double-blind manner.

### 2.8. Western blot analysis

On day 3 after SCI, long spinal cord segments (1.0 cm, in length) at the injury epicenter of rats (*n* = 5) in each group were dissected and immediately stored at -80°C for further Western blot analysis as previously published [42]. After protein extraction from the flap homogenate, the protein concentration was determined via a BCA test kit. 55 *μ*g protein was separated on a 12% (*w*/*v*) gel and transferred onto PVDF membranes. After blocking with 10% nonfat milk, the membranes were incubated with the following primary antibodies at 4°C overnight: mTOR (1 : 1000), p-mTOR (1 : 1000), p70S6K (1 : 1000), p-p70S6K (1 : 1000), LC3B (1 : 500), Beclin1 (1 : 1000), p62 (1 : 500), CTSD (1 : 500), ubiquitin (1:1000), PARP (1 : 1000), Bax (1 : 1000), cleaved CASP3 (1 : 400), cytochrome c (1 : 1000), and *β*-actin (1 : 1000). After that, the membranes were reincubated with the HRP-conjugated IgG secondary antibody (1 : 5000) at room temperature for 1.5 h. Then, bands on the membranes were visualized via the ECL-Plus Immune-Detection Kit. According to an established protocol [43], the intensity of the blot was quantified by densitometry using the C-DiGit*®* Blot Scanner and software (LI-COR Biosciences, USA) and was normalized to the *β*-actin band.

### 2.9. Immunofluorescence

On day 3 after SCI, after the aforementioned euthanization and perfusion for rats (*n* = 5) in every group, the cross-sections of rostral spinal cord segments (1.5 mm in length, 5 mm far from the epicenter) were prepared for immunofluorescence as we published [41]. After washing with PBS, the sections were blocked with 5% normal goat serum in PBS. Next, the sections were immune stained with primary antibodies overnight at 4°C: LC3B (1 : 200), NeuN (1 : 200), p62 (1 : 250), and cleaved CASP3 (1 : 100). Then, the slides were incubated with FITC-conjugated IgG secondary antibody (1 : 1000) or TRITC-conjugated IgG secondary antibody (1 : 100) for 1 h at 37°C and counterstained with DAPI staining solution. Next, the slides were visualized at 400x magnification under a fluorescent microscope (Nikon 90i, Japan), and neurons in the anterior horns were observed. In 10 slices per spinal cord segment, the absorbance values of LC3 II, p62 and cleaved CASP3 expressions were autocalculated and averaged in each group with ImageJ software (version 1.41.0, National Institutes of Health, USA).

### 2.10. Statistical analysis

Statistical analyses were carried out by using SPSS.19 software (Chicago, USA). All values are presented as the means ± standard error of the mean (SEM). Statistical evaluations of data were performed by repeated measures analysis of variance (ANOVA), followed by the LSD *post hoc* analysis (equal variances assumed) or Dunnett's T3 (equal variances not assumed) method. In all analyses, *p* values < 0.05 were considered statistically significant.

## 3. Results

### 3.1. Trehalose Promotes Autophagosome Recruitment in Neurons via an mTOR-Independent Pathway after SCI

**Figure 1:**
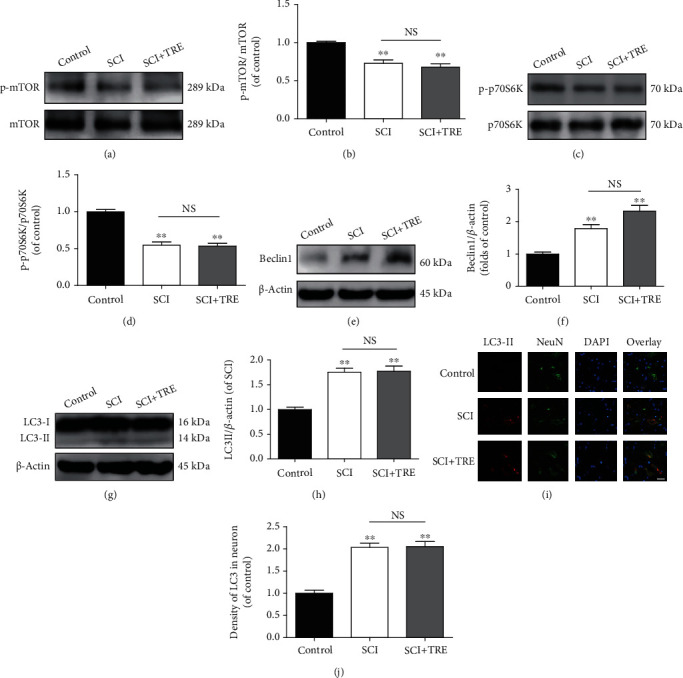
Trehalose promotes autophagosome recruitment in neurons via an mTOR-independent pathway after SCI. (a, c) Western blot analysis of the protein levels of p-mTOR/mTOR and p-p70S6K/p70S6K in the three different rat groups (control, SCI, and SCI+TRE) on day 3. (b, d) Quantitative analysis of the ratio of p-mTOR/mTOR and p-p70S6K/p70S6K in the three groups. (e, g) Western blot analysis of protein levels of Beclin1 and LC3II and in each group on day 3. (f, h) Quantitative analysis of Beclin1 and LC3II. (i) Immunostaining of LC3 in neurons in the lesions of the control, SCI, and SCI+TRE groups on day 3; scale bar: 40 *μ*m; (j) Quantitative analysis of LC3II density in neurons in the three groups. *N* = 5 in each group. NS stands for nonsignificance. ^∗∗^*P* < 0.01 for comparisons with the control rats.

First, we investigated the effect of trehalose on the mTOR pathway after SCI. Inhibition of the mTOR/p70 ribosomal protein S6 kinase (p70S6K) signaling pathway has been reported to play a crucial role in mTOR-dependent activation of autophagy. So, we investigated the expression of mTOR-dependent pathway-related proteins, p-mTOR/mTOR and p-p70S6K/p70S6K, in a spinal cord lesion on day3 after SCI. Quantitative analysis of western blotting showed that rates of p-mTOR/mTOR and p-p70S6K/p70S6K in the spinal cord lesion were significantly reduced in the untreated SCI group (72.8% and 54.7% of control, respectively, both *p* < 0.01; Figures 1(a)–1(d)). No significant differences of rates of p-mTOR/mTOR and p-70S6/70S6 were found between the SCI group and the SCI+TRE group (*p* = 0.37, *p* = 0.86; Figures 1(a)–1(d)). This finding suggests that trehalose treatment does not affect the mTOR pathway in SCI rats.

Next, we evaluated the effect of SCI on autophagosome recruitment and whether trehalose has an impact on this process. Here, we investigated whether autophagosome recruitment in the lesion is affected on day 3 after SCI. Compared with the control group, the untreated SCI group showed an obvious increase in the expression of Beclin1 (+78.9%, *p* < 0.01; Figures 1(e) and 1(f)), indicating increased formation of Beclin1-Vacuolar protein sorting 34- (Vps34-) and Vacuolar protein sorting 15- (Vps15-) core complexes, which are required in the preautophagosomal structure. Moreover, LC3II expression (a classical biomarker to quantify autophagosomes) in the untreated SCI group was significantly increased by 75.2% compared to that of the control group (*p* < 0.01; Figures 1(g) and 1(h)), and immunofluorescent staining also showed an increase of LC3II density in neurons in untreated SCI compared to the control group (+103.4%, *p* < 0.01; Figures 1(i) and 1(j)). These results indicate that the extent of autophagosome recruitment and formation was enhanced after SCI. Next, we investigated the effect of trehalose on autophagosome recruitment and formation in SCI on day 3. As hypothesized, in the SCI+TRE group, Beclin1 expression significantly increased by 30.3% compared to that of the untreated SCI group (*p* < 0.01; Figures 1(e) and 1(f)). This indicates that trehalose enhanced autophagosome recruitment in SCI rats. However, LC3II expression was not significantly increased in the TRE group compared with the untreated SCI group (*p* = 0.88; Figures 1(g) and 1(h)), and immunofluorescent staining also showed no significant change of LC3II density in neurons between the untreated SCI group and the SCI+TRE group (*p* > 0.05; Figures 1(i) and 1(j)). Together, these results showed that after SCI, trehalose enhanced autophagosome recruitment in neurons via an mTOR-independent pathway. However, the number of autophagosomes was not significantly changed after trehalose treatment, suggesting that trehalose may also increase the rate of autophagosome-lysosome fusion.

### 3.2. Trehalose Enhances Autophagy Activity in Neurons after SCI

To evaluate the effect of SCI and trehalose on autophagosome-lysosome fusion and overall autophagic protein degradation, we employed a number of markers to investigate autophagy flux (the dynamic process of autophagy) through the steps of the fusion of autophagosomes with lysosomes and its degradation of autophagy substrates [44], which is more accurate to reflect the overall activity of autophagy on day 3 after SCI. First, we investigated levels of p62 and ubiquitinated proteins, which are efficiently degraded through autophagy after p62/SQSTM1 binds LC3 and ubiquitinated proteins [45] and are commonly used to monitor autophagic substrate degradation [46]. We also measured the levels of cathepsin D (CTSD), which is the main lysosomal endopeptidase responsible for the degeneration of unwanted protein via autophagy. Both precursor (full length) and mature (cleaved) CTSD were evaluated.

**Figure 2:**
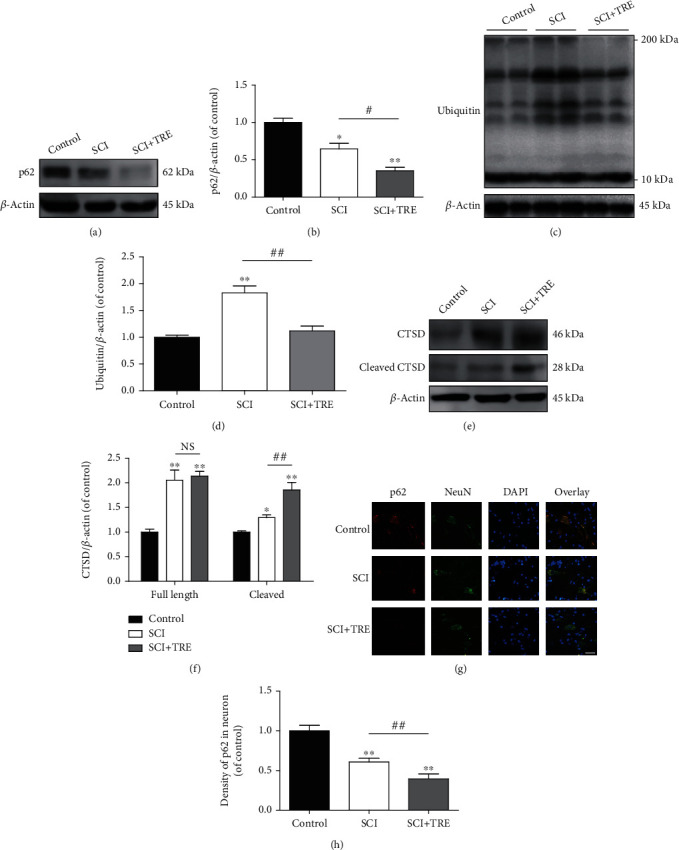
Effect of trehalose on autophagy flux in neurons after SCI. (a, c, and e) Western blot analysis of the protein levels of p62, cleaved CTSD/CTSD, and ubiquitin in the three different rat groups (control, SCI, and SCI+TRE) on day 3. (b, d, and f) Quantitative analysis of p62, cleaved CTSD, CTSD, and ubiquitin in the three groups. (g) Immunostaining of p62 in neurons in the lesion of the three rat groups on day 3; scale bar: 40 *μ*m; (h) Quantitative analysis of p62 density in neurons. *N* = 5 in each group. ^∗^*P* < 0.05 and ^∗∗^*P* < 0.01 for comparisons with the control rats, ^#^*P* < 0.05 and ^##^*P* < 0.01 for comparisons between SCI rats.

Quantitative analysis of Western blots demonstrated that compared with the control group, the expression of p62 in the untreated SCI group was significantly decreased (-35.53%, *p* < 0.01; Figures 2(a) and 2(b)), whereas ubiquitinated protein expression was significantly increased (+82.84%, *p* < 0.01; Figures 2(c) and 2(d)). In addition, SCI induced significant increases of both cleaved CTSD (+29.3%, *p* < 0.05) and full length CTSD (+105.3%, *p* < 0.01) compared to the control group (Figures 2(e) and 2(f)). Immunofluorescence showed a decrease of p62 density in neurons in the lesion of untreated SCI (-39.01%, *p* < 0.01; Figures 2(g) and 2(h)) compared to the control group. These results indicate that while the amount of proteins degraded via autophagy was likely increased following SCI (consistent with decreased p62 levels) and that the neurons had an increased capacity to degrade proteins (consistent with increased cleaved CTSD), the substrate burden may be elevated beyond this increased capacity to degrade proteins (consistent with increased ubiquitinated proteins).

In contrast with untreated conditions, trehalose treatment led to significantly decreased p62 and ubiquitinated proteins in the SCI+TRE group compared to the untreated SCI group (-45.3%, *p* < 0.01; and -38.90%, *p* < 0.01; respectively. Figures 2(a)–2(d)). Furthermore, trehalose led to a significant increase of cleaved CTSD compared to the untreated SCI group (+43.6%, *p* < 0.01), but no significant change in full length CTSD (*p* > 0.05; Figures 2(e) and 2(f)). Immunofluorescent staining of p62 also showed that the density of p62 in neurons in the lesion of the SCI+TRE group was decreased to 63.91% of the untreated SCI group (*p* < 0.01; Figures 2(g) and 2(h)). Overall, these results indicate that trehalose enhanced autophagic protein degradation following SCI in rats, which may suggest an increase in autophagy flux.

### 3.3. Trehalose Inhibits Apoptosis via the Intrinsic Mitochondria-Dependent Pathway in SCI Rats

**Figure 3:**
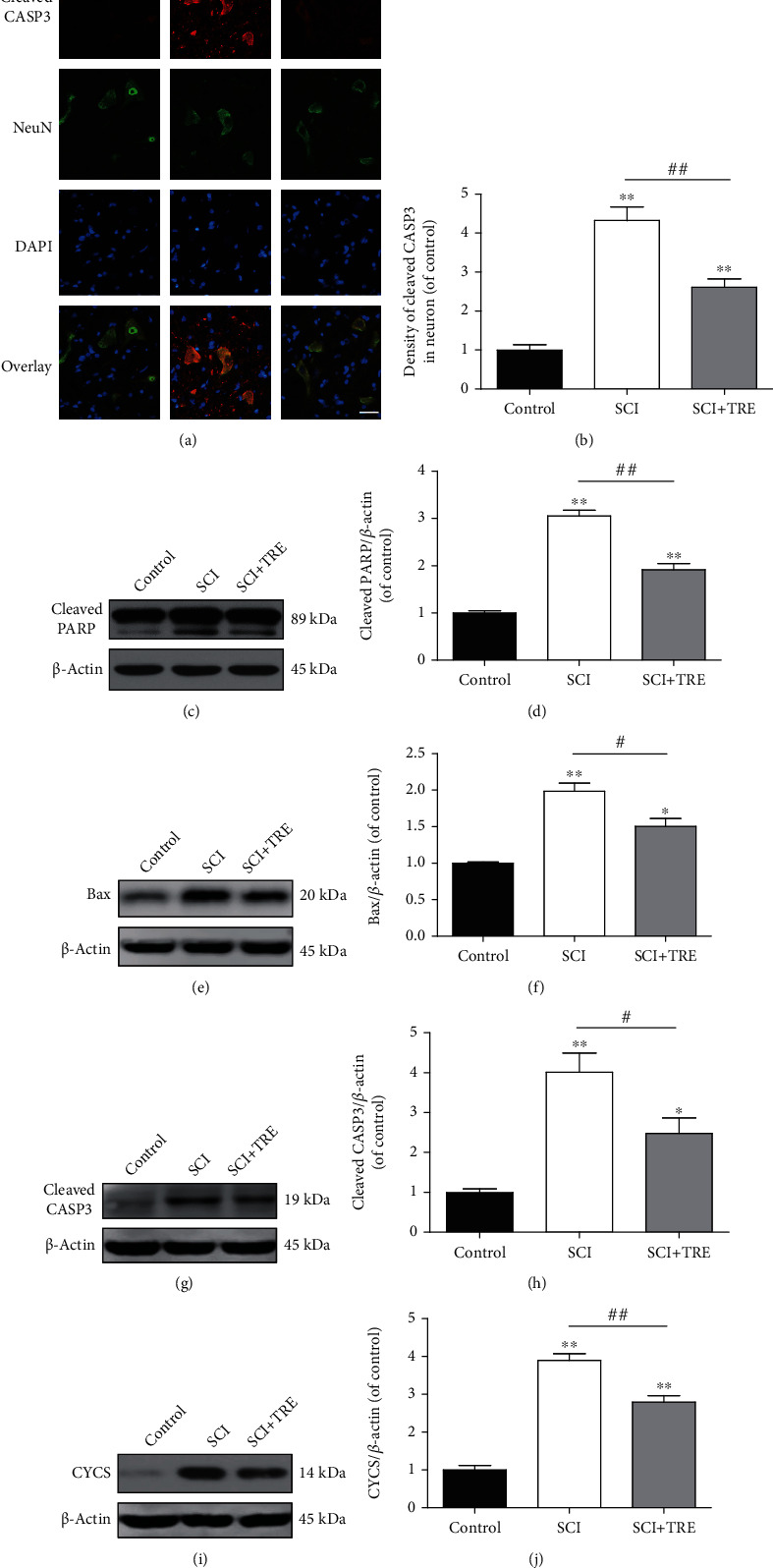
Effect of trehalose on mitochondrial damage-induced apoptosis in neurons after SCI. (a) Immunostaining of cleaved CASP3 in neurons in the lesion of the three different groups (control, SCI, and SCI+TRE) on day 3; scale bar: 40 *μ*m; (b) Quantitative analysis of cleaved CASP3 density in neurons of each group. (c, e, g, and i) Western blot analysis of the protein levels of cleaved PARP, Bax, cleaved CASP3, and CYCS in each group on day 3. (d, f, h, and j) Quantitative analysis of cleaved PARP, Bax, cleaved CASP3, and CYCS in the three groups. *N* = 5 in each group. ^∗^*P* < 0.05 and ^∗∗^*P* < 0.01 for comparisons with the control rats, ^#^*P* < 0.05 and ^##^*P* < 0.01 for comparisons between SCI rats.

During the secondary injury phase of SCI, autophagy is thought to be an effective pathway to reduce mitochondria-dependent apoptosis via enhanced elimination of damaged mitochondria (mitophagy) [18]. Cytochrome c (CYCS) forms a complex with Apaf-1 and an inactive preform of caspase, caspase-9, which in turn can trigger a cascade by activating caspase-3 (CASP3), ultimately executing apoptosis. Bax induces cytochrome c release. Poly(ADP-ribose) polymerase (PARP) activation promotes CYCS. To further evaluate the effect of SCI and trehalose on apoptosis, protein expressions of cleaved polyPARP, cleaved CASP3, Bax, and CYCS were examined on day 3. Immunofluorescent staining showed a significant increase of cleaved CASP3 density in neurons in the lesion of untreated SCI (+332.15%, *p* < 0.01; Figures 3(a) and 3(b)) compared to the control group. Quantitative analysis of Western blots also demonstrated that compared with the control group, the expressions of cleaved PARP and Bax were increased by 205.59% and 98.71%, respectively, in the untreated SCI group (both *p* < 0.01; Figures 3(c)–3(f)). Western blotting results also revealed that SCI induced significant increases of both cleaved CASP3 (+301.04%, *p* < 0.05) and CYCS (+290.32%, *p* < 0.01), compared to the control group (Figures 2(e) and 2(f)). These findings suggest that intrinsic mitochondria-dependent apoptosis was significantly enhanced after SCI.

Despite significant increases in mitochondria-dependent apoptosis due to SCI, administration of trehalose exerts a profound regulatory effect on mitochondria-dependent apoptosis. As shown in Figures 3(a) and 3(b), the density of CASP3 immunostaining in neurons in the lesion of the SCI+TRE group was reduced to 60.40% compared with the untreated SCI group (*p* < 0.01). Quantitative analysis of western blotting results showed that trehalose administration reduced the expressions of cleaved PARP and Bax to 67.75% and 75.74%, respectively, as compared with the untreated SCI group (both *p* < 0.01; Figures 3(c)–3(f)). Western blotting also showed that trehalose treatment reduced the level of cleaved CASP3 to 61.72% compared with the untreated SCI group (*p* < 0.01; Figures 3(g) and 3(h)). Furthermore, SCI+TRE rats showed a significant decrease of CYCS expression compared with the untreated SCI group (-28.33%, *p* < 0.01; Figures 3(i) and 3(j)). However, while trehalose profoundly reduced the activation of mitochondria-dependent apoptosis due to SCI, the level of apoptosis was still higher than the control (all markers *p* < 0.05; Figures 3(a)–3(j)). Together, these data suggest that trehalose substantially inhibited mitochondria-dependent apoptosis due to SCI in neurons.

### 3.4. Trehalose Reduces Lesion Cavity Expansion, Decreases Neuron Loss, and Improves Functional Recovery in SCI Rats

**Figure 4:**
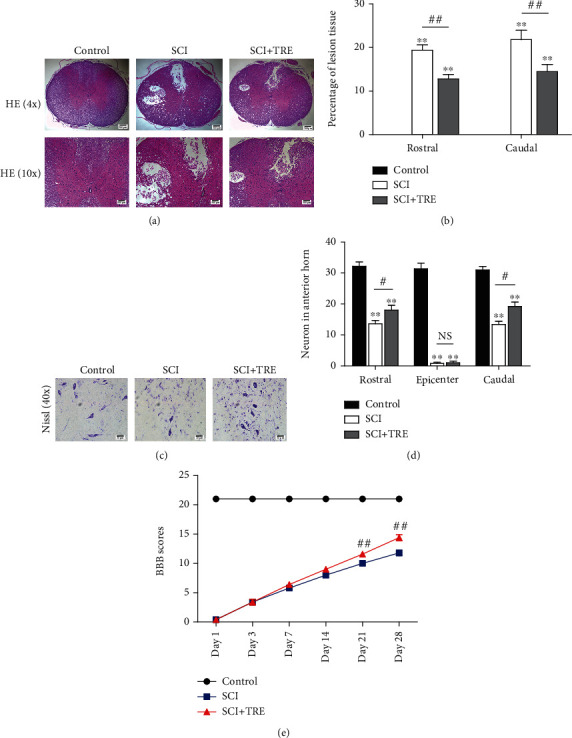
Effect of trehalose on tissue preservation, neuron survival, and functional recovery in SCI rats. (a) HE staining of the rostral segment (5 mm to epicenter) of the injured spinal cord in the three different groups (control, SCI, and SCI+TRE) on day 7; scale bar: 200 *μ*m and 100 *μ*m. (b) Quantitative analysis of cavity percentage in the injured spinal cord at the rostral and caudal segments (5 mm to epicenter). (c) Nissl staining of the rostral segment (5 mm to epicenter) of the injured spinal cord in each group on day 7; scale bar: 20 *μ*m. (d) Quantitation of the number of neurons in the injured spinal cord at the epicenter, rostral, and caudal segments. (e) BBB scores of rats in the control group, the SCI group, and the SCI+TRE group at 1, 3, 7, 14, 21, and 28 days after acute traumatic SCI. *N* = 5 for HE staining, Nissl staining, and BBB score testing. NS stands for nonsignificance. ^∗∗^*P* < 0.01 for comparisons with the control rats and ^#^*P* < 0.05 and ^##^*P* < 0.01 for comparisons between SCI rats.

To determine the effect of trehalose on neuron preservation and survival, we performed HE staining to evaluate tissue damage and Nissl staining to examine neuron survival in following SCI in rats. HE staining at day 7 showed that both the untreated SCI group and the SCI+TRE group presented severe damage with cavity formation in dorsal white matter and central grey matter (Figures 4(a) and 4(b)). To quantify the extent of damage, the percentage of tissue cavity in the injured spinal cord segment was calculated. We found that there was significantly lower cavity percentage in the SCI+TRE rats compared with the untreated SCI rats at the rostral and caudal segments (5 mm to epicenter; 12.80 ± 2.28% vs. 19.40 ± 2.20% and 14.50 ± 3.59% vs. 21.82 ± 4.78%, respectively; both *p* < 0.01; Figures 4(a) and 4(b)). Moreover, Nissl staining and enumeration of neurons showed that there was a significant loss of neurons on day 7 in the untreated SCI group compared with the control group at the rostral and caudal segments (32.20 ± 3.11 vs. 13.60 ± 2.41 and 30.00 ± 2.55 vs. 13.40 ± 2.30, respectively; both *p* < 0.01; Figures 4(c) and 4(d)). With trehalose administration, the number of surviving neurons increased to 18.00 ± 3.54 at the rostral segment and 19.20 ± 3.19 at the caudal segment (both *p* < 0.05, compared to the untreated SCI group). To explore whether trehalose treatment can influence locomotor functional recovery, we measured BBB scores for 4 weeks. The primary BBB scores in the control, TRE, and SCI+TRE groups are presented in Supplementary Table 1. The results showed that the control group featured normal BBB scores of 21 points, and the untreated SCI group and the SCI+TRE group were both subnormal throughout the entire experimental period. Although no significant differences in BBB score were detected between the SCI and SCI+TRE groups 1, 3, 7, and 14 days after SCI (*p* = 1, *p* = 1, *p* = 0.126, and *p* = 0.077; Figure 4(e)), respectively, the mean BBB score in the SCI+ TRE group was higher than that in the SCI group on days 7 and 14. Furthermore, BBB scores of the SCI+TRE group were significantly higher than those of the untreated SCI group at 21 and 28 days after SCI (both *p* < 0.01; Figure 4(e)). These results suggest that trehalose administration promotes tissue preservation and neuron survival, ultimately leading to improved functional recovery.

### 3.5. Trehalose's Autophagy Activating Effects Are Inhibited by Autophagy Flux Inhibitor CQ

**Figure 5:**
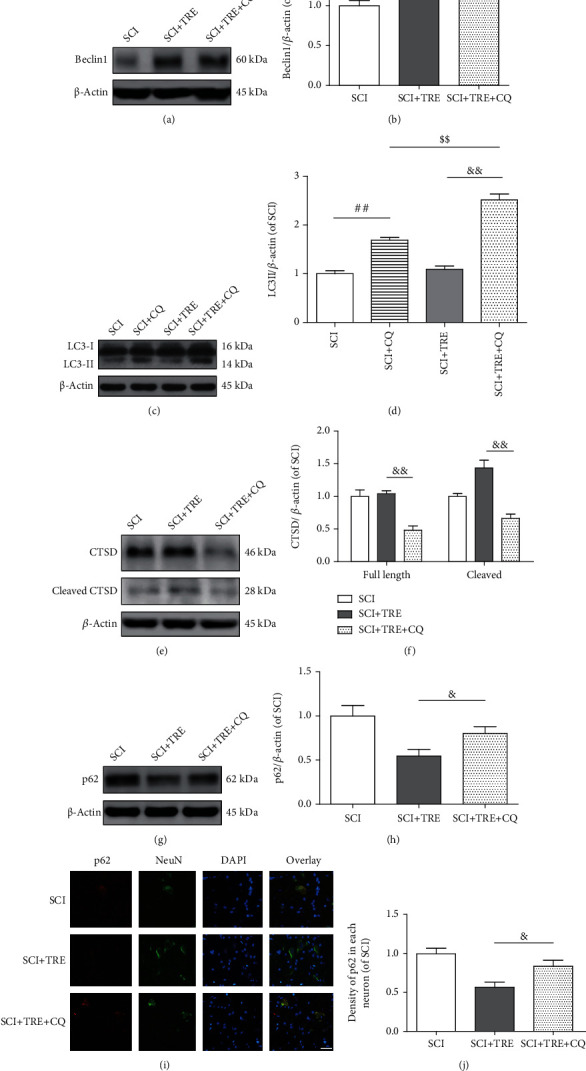
Effect of chloroquine (CQ) on trehalose's boost of autophagy flux in SCI rats. (a, c) Western blot analysis of the protein levels of Beclin1 and LC3II in the three different rat groups (SCI, SCI+TRE, and SCI+TRE+CQ) on day 3. (b, d) Quantitative analysis of the levels of Beclin1 and LC3II. (e, g) Western blot analysis of the protein levels of CTSD, cleaved CTSD, and p62 in the three different rat groups on day 3. (f, h) Quantitative analysis of the levels of CTSD, p-CTSD, and p62. (i) Immunostaining of p62 in neurons of the SCI, SCI+TRE, and SCI+TRE+CQ groups on day 3; scale bar: 40 *μ*m. (j) Quantitative analysis of p62 density in each group. *N* = 5. NS stands for nonsignificance. ^##^*P* < 0.01 for comparisons between SCI rats, ^$$^*P* < 0.01 for comparisons between the SCI+CQ rats, and ^&^*P* < 0.05 and ^&&^*P* < 0.01 for comparisons between the SCI+TRE rats.

To confirm that the beneficial effects of trehalose in SCI recovery are due to the upregulation of autophagy processes, CQ, a classical autophagy flux inhibitor via inhibition of lysosomal acidification, was used to treat TRE+SCI rats on day 3 after SCI. First, Western blot analysis revealed no significant difference of protein expression of Beclin1 between the SCI+TRE group and the SCI+TRE+CQ group (*p* = 0.997; Figures 5(a) and 5(b)). This confirmed that CQ did not affect autophagosome recruitment in SCI following trehalose administration. Next, pharmacological activity of CQ in deactivating lysosomes and blocking autophagy flux was assessed. Western blot analysis showed a significant 131.01% upregulation of LC3II in the SCI+TRE+CQ group compared with the SCI+TRE group, and the turnover of LC3II in SCI+TRE rats is higher than that of SCI rats (*p* < 0.01, *p* < 0.01; Figures 5(c) and 5(d)). For CTSD and ubiquitinated protein quantitative analysis, CQ administration led to significant reductions of cleaved CTSD (-53.82%, *p* < 0.01; Figures 5(e) and 5(f)) and full length CTSD (-53.90%, *p* < 0.01; Figures 5(e) and 5(f). Furthermore, p62 levels in SCI+TRE+CQ rats were significantly increased compared to SCI+TRE rats (+46.92%; Figures 5(g) and 5(h)), and the density of p62 immunostaining in neurons in the lesion of the SCI+TRE+CQ group also increased 39.23% compared with the SCI+TRE group (Figures 5(i) and 5(j)). Together, these results suggest that CQ restrained trehalose's effective enhancement of autophagy via blocking autophagy flux, both confirming that autophagy activation may underlie the therapeutic effects of trehalose and reinforcing the likelihood that trehalose enhances autophagy flux after SCI.

### 3.6. Trehalose's Inhibition of Mitochondria-Dependent Apoptosis Is Abolished by Blocking Autophagy Flux

**Figure 6:**
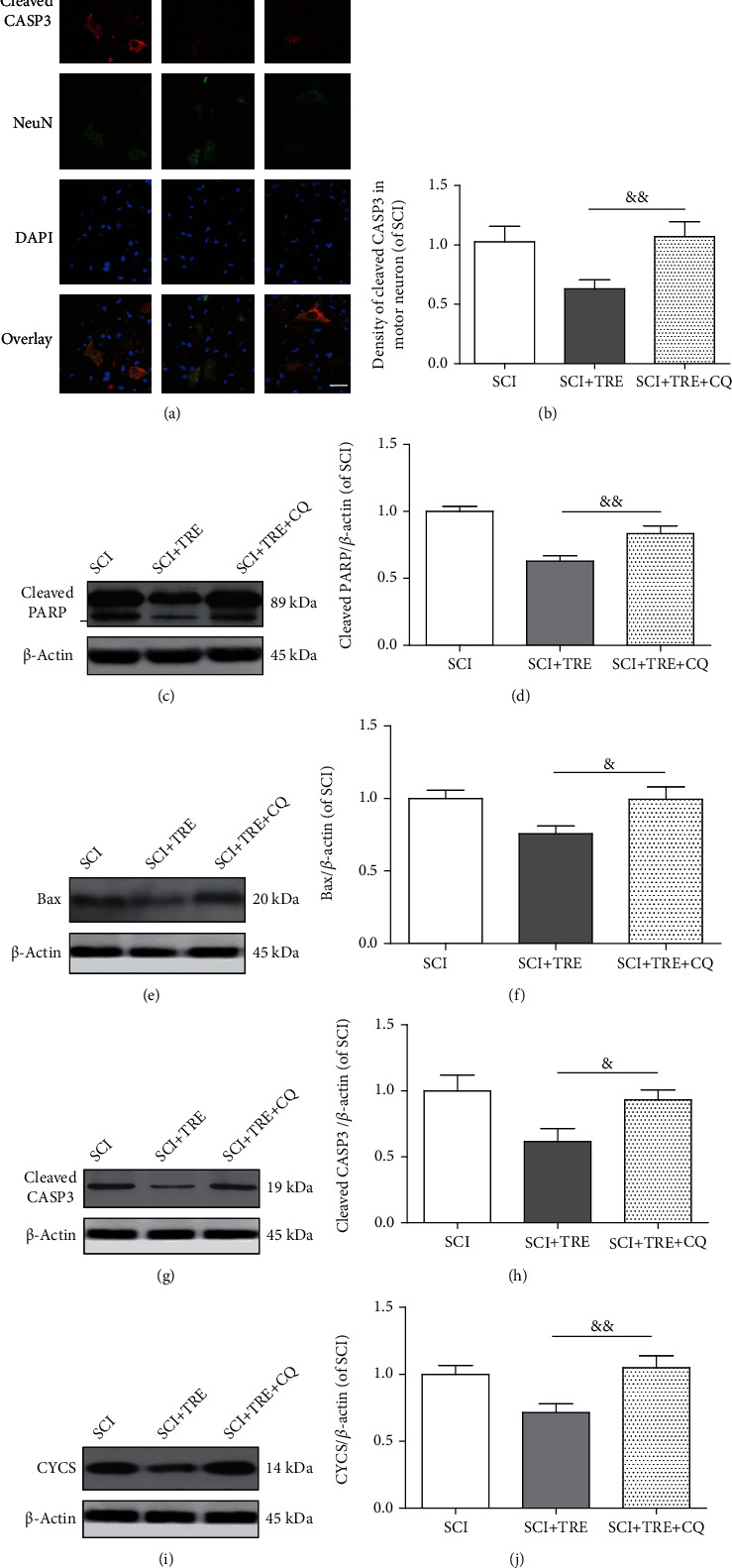
Effect of CQ on trehalose's inhibition of mitochondrial damage-induced apoptosis in SCI rats. (a) Immunostaining of cleaved CASP3 in neurons in the lesion of the three rat groups (SCI, SCI+TRE, and SCI+TRE+CQ) on day 3; scale bar: 40 *μ*m. (b) Quantitative analysis of cleaved CASP3 density in neurons of the three different groups. (c, e, g, and i) Western blot analysis of the protein levels of cleaved PARP, Bax, cleaved CASP3, and CYCS in each group on day 3. (d, f, h, and j) Quantitative analysis of cleaved PARP, Bax, cleaved CASP3, and CYCS in the three groups. *N* = 5. ^&^*P* < 0.05 and ^&&^*P* < 0.01 for comparisons between the SCI+TRE rats.

We further evaluated mitochondria-dependent apoptosis-related proteins in trehalose-treated SCI rats with additional CQ administration on day 3. As shown in Figures 6(a) and 6(b), the density of cleaved CASP3 immunostaining in neurons in the lesion of the SCI+TRE+CQ group was increased by 74.32% compared to the SCI+TRE group (*p* < 0.01). Quantitative analysis of western blotting results also demonstrated that CQ upregulated the expressions of cleaved PARP and Bax of the SCI+TRE group by 33.01% and 31.27%, respectively (both *p* < 0.01; Figures 6(c)–6(f)). In addition, as shown in Figures 4(g) and 4(h), compared with the SCI+TRE group, CQ treatment upregulated the level of cleaved CASP3 of the SCI+TRE+CQ group by 50.96% (*p* < 0.01). Furthermore, the SCI+TRE+CQ group showed significantly increased CYCS expression compared with the SCI+TRE group (+46.77%, *p* < 0.01; Figures 6(i) and 6(j)). These data show that CQ abolished trehalose's inhibition of mitochondria-dependent apoptosis, and that trehalose's anti-apoptotic effect might be due to mTOR-independent activation of autophagy flux.

### 3.7. Neuron Survival Promotion and Tissue Preservation by Trehalose Therapy Are Inhibited by Autophagy Inhibition

**Figure 7:**
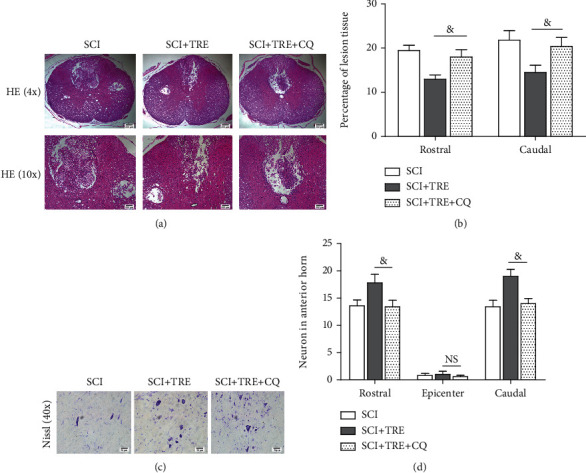
Effect of CQ on trehalose's promotion of neuron survival and tissue preservation in SCI rats. (a) HE staining of the rostral segment (5 mm to epicenter) of the injured spinal cord in the three different groups (SCI, SCI+TRE, and SCI+TRE+CQ) on day 7; scale bar: 200 *μ*m and 100 *μ*m. (b) Quantitative analysis of cavity percentage in the injured spinal cord at the rostral and caudal segments (5 mm to epicenter). (c) Nissl staining of the rostral segment (5 mm to epicenter) of the injured spinal cord in each group on day 7; scale bar: 20 *μ*m. (d) Quantitation of the number of neurons in the injured spinal cord at the epicenter, rostral, and caudal segments. *N* = 5. ^&^*P* < 0.05 and ^&&^*P* < 0.01 for comparisons between SCI+TRE rats.

HE staining revealed that CQ treatment substantially ablated the neuroprotective effects of trehalose in terms of damage to dorsal white matter and central grey matter in SCI+TRE rats (Figure 7(a)). To quantify the damage extent, the percentage of tissue cavity in the injured spinal cord segment was calculated on day 7. The cavity burden in the SCI+TRE+CQ rats was significantly increased compared with that in the SCI+TRE rats at the rostral and caudal segments (5 mm to epicenter), respectively (18.02 ± 3.68% vs. 12.80 ± 2.28% and 20.39 ± 4.45% vs. 14.50 ± 3.59%; both *p* < 0.05; Figures 7(a) and 7(b)), and measurements approximated the untreated SCI group (all *p* > 0.05). Nissl staining results also revealed that CQ administration decreased the number of surviving neurons to 13.40 ± 2.70 at the rostral segment and 14.00 ± 2.12 at the caudal segment, as compared with the SCI+TRE groups (both *p* < 0.01; Figures 7(c) and 7(d)), which again approximated the untreated SCI group (all *p* > 0.05). Altogether, these results showed that CQ inhibited trehalose's effective promotion of neuron survival in the injured spinal cord likely via abolishing trehalose's enhancement of autophagy and inhibition of mitochondria-dependent apoptosis, suggesting that trehalose's neuroprotective and neuroregenerative effects may be due in part to inhibition of mitochondria-dependent autophagy.

## 4. Discussion

Using trehalose, we showed for the first time that mTOR-independent activation of autophagy clearly plays a crucial protective role in acute SCI, and that augmenting autophagy via trehalose significantly improves outcomes. More specifically, we found that trehalose suppresses the expansion of cavitary lesions in nerve tissue, inhibits neuron loss, and improves functional recovery in SCI rats. Mechanistically, our results showed that the neuroprotective effects of trehalose are due to the activation of autophagy flux and subsequent inhibition of mitochondria-dependent apoptosis. Our results provide crucial insight into the previously unknown role of autophagy in SCI.

Autophagy is a key cellular pathway that plays a crucial role in cellular survival under stress conditions, and it is one of the major catabolic mechanisms in eukaryotes for the degradation of cytoplasmic constituents through the autophagosomal-lysosomal pathway [47]. Previously, many landmark studies have shown that autophagy plays a significant role in chronic CNS disorders such as ALS, Huntington's disease, and Alzheimer's dementia. Findings from these studies have sparked considerable interest into investigating the role of autophagy in acute CNS injuries, such as SCI, and a growing body of evidence suggests that autophagy plays a critical role in SCI as well [16, 18]. However, results have been largely controversial, in part due to the limitations of mTOR-dependent activation of autophagy using rapamycin, which is known to have various effects on various nonautophagy cellular pathways important in acute injury, such as angiogenesis [5, 48]. Thus, the role of autophagy in acute SCI and whether increasing autophagy is beneficial remained largely unknown, requiring further investigation using mTOR-independent autophagy agonists. This controversy may be due to rapamycin's adverse effects on angiogenesis via the mTOR pathway. Herein, trehalose, an mTOR-independent autophagy enhancer, was administered to elucidate the effect of autophagy activation in TSCI and subsequent recovery. Of note, while recent reports have shown that trehalose may improve outcomes following SCI, whether these benefits are related to autophagy is unknown [31–34].

Autophagy differentially affects TSCI depending on the type and severity and appears to contribute to differences in outcomes regarding autophagy flux [10], which seems to be inhibited after severe traumatic injury and enhanced after relatively moderate injury. In the present study, we found that SCI leads to increased autophagosome recruitment and formation, which supports that autophagy flux is enhanced in moderate SCI, and decreased autophagosome-lysosome fusion, and that with administration of trehalose, both autophagosome recruitment and autophagosome-lysosome fusion are further augmented.

Interestingly, the number of autophagosomes was not significantly changed with trehalose treatment in SCI, which may indicate that the rate of autophagosome fusion with lysosomes was enhanced with trehalose in SCI. Our results showed that this is indeed the case given that with coadministration of chloroquine (CQ), a classical autophagy flux inhibitor via suppressing lysosomotropic acid [49], the level of LC3II turnover in the SCI+TRE group is significantly higher than that in the untreated SCI group (Figures 5(c), and 5(d)), suggesting that the unchanged LC3II after administration with trehalose alone was likely due to the increased rate of autophagosome-lysosome fusion. Together, these findings clearly demonstrate that autophagy flux is increased in SCI, and further augmented with trehalose treatment.

In the secondary injury phase of SCI, mitochondrial dysfunction is believed to play a critical role in neuron death by increasing generation of reactive oxygen species (ROS) and subsequently initiating apoptosis [50–52]. In SCI, the release of glutamate and activation of glutamate receptors lead to overaccumulation of intracellular Ca^2+^, which can further result in overgeneration of ROS [53]. ROS overproduction increases the catabolism of membrane phospholipids, promotes the generation of lipid peroxides [54], and further causes mitochondrial dysfunction via damaging its membranes [55]. Subsequently, mitochondrial dysfunction can result in further increases in ROS [53]. Thus, the vicious cycle of ROS overgeneration destroys the integrity of mitochondria, causing increased mitochondrial injury and ultimately leading to mitochondrial damage-induced apoptosis [56, 57].

To evaluate the intensity of mitochondrial damage-induced apoptosis, several marker proteins can be measured, such as cytochrome c, caspase-3, Bax, and PARP. In brief, early in the process, cytochrome c, a key component of the electron transport chain, is released into the cytosol and complexes with Apaf-1 and an inactive preform of caspase, caspase-9 [58]. In the presence of dATP or ATP, this complex processes and activates caspase-9, which in turn can trigger a cascade by activating other caspases, such as caspase-3, caspase-6, and caspase-7, ultimately executing apoptosis [59]. Furthermore, Bax is a proapoptotic Bcl-2-family protein, which induces cytochrome c release and caspase activation [58]. In the event of increased oxidative stress and DNA damage, poly(ADP-ribose) polymerase (PARP) activation in the nucleus leads to the formation of poly(ADP-ribose) (PAR) in large amounts and NAD+ depletion, triggers the release of mitochondrial AIF, and further promotes cytochrome c release from mitochondria and subsequent caspase activation [60]. In the present study, analyses of both immunofluorescence staining and western blotting results revealed that cytochrome c, cleaved caspase-3, Bax, and cleaved PARP were all significantly increased in neurons following SCI. Strikingly, after the administration of trehalose, these enhanced markers were obviously depressed, indicating that while SCI leads to increased mitochondria-dependent apoptosis, treatment with trehalose inhibits this process with improved neuron survival and reduced cavity burden. TUNEL staining is a useful marker to valuate apoptosis level after SCI and may be considered in future investigations of trehalose's effects in SCI. In our previous SCI study [5], the result trend between TUNEL staining and caspase-3 expression in groups were the same.

Of note, a difference in autophagy markers may result from apoptosis. Thus, we used the autophagy inhibitor CQ to assess and exclude this possibility, and our results showed that CQ reversed the majority of trehalose's therapeutic effect. This suggests that most of the changes of autophagy markers were likely due to changes in autophagy flux, and changes in apoptosis were likely a result of autophagy, not vice versa. It has been reported that trehalose exerts a protective effect on mitochondria in various disease models such as amyotrophic lateral sclerosis [15], osteoarthritis [61], and acute kidney injury [62]. Mechanistically, trehalose strengthens mitochondrial structural integrity and attenuates dysfunctions of membrane potential, respiratory chain, and oxidative phosphorylation. Currently, there are no detailed reports on the effects of trehalose on mitochondria stability in SCI. Therefore, future studies including transmission electron microscope (TEM) examination for mitochondria structure and assessment of mitochondrial functions following trehalose administration in SCI are warranted for further insight into trehalose's mechanism of action in SCI.

To determine whether trehalose's inhibition of mitochondria-dependent apoptosis is mechanistically linked to the enhancement of autophagy flux, CQ, a potent inhibitor of autophagy flux [49], was coadministered with trehalose, and markers of autophagy and mitochondria-dependent apoptosis were revisited. Results of Figures 5, 6 and 7 together showed that trehalose's effect on activating autophagy is reduced with CQ coadministration, and that trehalose's suppression of mitochondria-dependent apoptosis was significantly abolished, which led to subsequently worsened recovery. Together, these results showed mechanistically that trehalose's inhibition of mitochondria-dependent apoptosis is closely tied to the activation of autophagy flux. Furthermore, our results showed that trehalose promotes functional recovery, reduces cavitary lesion burdens, and improves neuron survival. These beneficial effects were also ablated with CQ coadministration, suggesting that both autophagy and mitochondria-dependent apoptosis play crucial roles in SCI and contribute to the neuroprotective properties of trehalose.

Autophagy and mitochondria-dependent apoptosis have been shown to be closely intertwined. Generally, autophagy is considered to be a nonspecific process that eliminates various substrates such as damaged organelles, toxic agents, and long-lived proteins [63]. However, autophagy occasionally selects specific organelles, such as in “mitophagy,” where cells actively remove mitochondria [64]. Mitophagy is a precise mechanism that regulates mitochondrial number in response to the metabolic demand of a cell and also as a form of quality control to eliminate dysfunctional mitochondria [65]. It has been reported previously that mitophagy is upregulated in the event of acute CNS injuries such as SCI, which may play a protective role during the process of secondary injury phase [66]. Therefore, trehalose's repression of mitochondria-dependent apoptosis may be associated with its promotion of mitophagy in SCI. Future studies using the analysis of mitophagy markers such as Bcl-2/E1B-19K-interacting protein3 (BNIP3) [67] and Nip-like protein X (NIX) [68] in combination with transmission electron microscopy (TEM) observation for mitochondria are necessary to thoroughly uncover the mechanistic links between mTOR-independent activation of autophagy and inhibition of mitochondria-dependent apoptosis in the setting of acute CNS injuries such as SCI.

## 5. Conclusion

We showed for the first time that mTOR-independent activation of autophagy via trehalose following SCI attenuates mitochondria-dependent apoptosis, promotes neuron survival, and improves functional outcomes. This is novel evidence supporting the hypothesis that autophagy plays a critical neuroprotective role in SCI. The results from this study indicate that the reactive strengthening of autophagy is an important pathophysiological event in an acute injury, and that mTOR-independent autophagy agonists may be promising novel therapeutics for SCI.

## Data Availability

All data supporting the conclusions of this manuscript are provided in the text and figures. Please contact the author for data requests.

## Supplementary Materials

Supplementary Materials Supplementary Table 1: primary BBB scores in the Control, TRE, and SCI+TRE groups.
