# Supplementary material for: Trehalose Augments Neuron Survival and Improves Recovery from Spinal Cord Injury via mTOR-Independent Activation of Autophagy
Source: Oxid Med Cell Longev. 2021 Jul 10;2021:8898996. doi: 10.1155/2021/8898996 (PMC8289614; doi:10.1155/2021/8898996)
Supplement: Supplementary Materials — Supplementary Table 1: primary BBB scores in the Control, TRE, and SCI+TRE groups. [file 8898996.f1.docx]

**Supplementary Information**

**for**

**Trehalose Augments Neuron Survival and Improves Recovery from Spinal Cord Injury via mTOR-Independent Activation of Autophagy**

Kailiang Zhou, MD, PhD^1,2^, Huanwen Chen, BA ^2^, Huazi Xu, MD^1^, Xiaofeng Jia, MD, PhD ^2,3,4,5,6^

|  | Control | SCI | SCI+TRE |
| --- | --- | --- | --- |
| Day 1 | 21 | 0 | 1 |
|  | 21 | 1 | 0 |
|  | 21 | 1 | 0 |
|  | 21 | 0 | 1 |
|  | 21 | 0 | 0 |
| Day 3 | 21 | 3 | 2 |
|  | 21 | 3 | 3 |
|  | 21 | 5 | 5 |
|  | 21 | 3 | 4 |
|  | 21 | 3 | 3 |
| Day 7 | 21 | 5 | 6 |
|  | 21 | 5 | 6 |
|  | 21 | 6 | 7 |
|  | 21 | 7 | 7 |
|  | 21 | 6 | 6 |
| Day 14 | 21 | 7 | 10 |
|  | 21 | 7 | 9 |
|  | 21 | 9 | 8 |
|  | 21 | 9 | 10 |
|  | 21 | 8 | 8 |
| Day 21 | 21 | 9 | 11 |
|  | 21 | 10 | 12 |
|  | 21 | 10 | 11 |
|  | 21 | 11 | 13 |
|  | 21 | 10 | 11 |
| Day 28 | 21 | 10 | 14 |
|  | 21 | 12 | 15 |
|  | 21 | 12 | 13 |
|  | 21 | 13 | 16 |
|  | 21 | 12 | 14 |

Supplementary Table 1. Primary BBB scores in the Control, TRE, and SCI+TRE groups.
